# Emotional-Health-Oriented Urban Design: A Novel Collaborative Deep Learning Framework for Real-Time Landscape Assessment by Integrating Facial Expression Recognition and Pixel-Level Semantic Segmentation

**DOI:** 10.3390/ijerph192013308

**Published:** 2022-10-15

**Authors:** Xuan Zhang, Haoying Han, Lin Qiao, Jingwei Zhuang, Ziming Ren, Yang Su, Yiping Xia

**Affiliations:** 1 Institute of Urban and Rural Planning Theories and Technologies, College of Civil Engineering and Architecture, Zhejiang University, Hangzhou 310058, China; 2 Institute of Landscape Architecture, College of Agriculture and Biotechnology, Zhejiang University, Hangzhou 310058, China; 3 Department of Landscape Architecture, School of Civil Engineering and Architecture, Zhejiang Sci-Tech University, Hangzhou 310018, China; 4 The Architectural Design & Research Institute of Zhejiang University Co., Ltd., Hangzhou 310030, China

**Keywords:** urban green space, landscape assessment, deep learning, facial expression recognition, semantic segmentation

## Abstract

Emotional responses are significant for understanding public perceptions of urban green space (UGS) and can be used to inform proposals for optimal urban design strategies to enhance public emotional health in the times of COVID-19. However, most empirical studies fail to consider emotion-oriented landscape assessments under dynamic perspectives despite the fact that individually observed sceneries alter with angle. To close this gap, a real-time sentimental-based landscape assessment framework is developed, integrating facial expression recognition with semantic segmentation of changing landscapes. Furthermore, a case study using panoramic videos converted from Google Street View images to simulate changing scenes was used to test the viability of this framework, resulting in five million big data points. The result of this study shows that through the collaboration of deep learning algorithms, finer visual variables were classified, subtle emotional responses were tracked, and better regression results for valence and arousal were obtained. Among all the predictors, the proportion of grass was the most significant predictor for emotional perception. The proposed framework is adaptable and human-centric, and it enables the instantaneous emotional perception of the built environment by the general public as a feedback survey tool to aid urban planners in creating UGS that promote emotional well-being.

## 1. Introduction

During the COVID-19 pandemic, almost all countries imposed strict regulations, such as social distancing and movement restrictions [1,2], which had a negative effect on mental health, leading to symptoms such as depression and anxiety [3,4]. According to a large number of empirical studies, exposure to urban green space (UGS) can contribute to promoting the physical, psychological, emotional, and mental health of urban residents [5,6,7] and help people perceive more positive moods and cope with tough situations [8,9]. Natural elements, such as grass [10], trees [11], bodies of water [12], and sky [13] in urban environments have restorative potential to promote positive emotions and have the power to improve urban settings. However, few studies have explored the emotional evaluation of human-viewed UGSs in a dynamic process; thus, we have little understanding of how the changing visual variables of UGS relate to aesthetic emotions and thus affect the public’s mental status. For the time being, it is crucial to develop a framework for real-time emotive landscape assessment in order to better understand how the public emotionally responds to UGS and to create appropriate planning and design strategies that will optimize their benefits to quality of life.

Aesthetic emotions reflect subjective aesthetic judgement, which is a major predictor for public appreciation of the aesthetic appeal of UGS [14,15]. Given the importance of aesthetic emotions, measuring people’s dynamic emotional perception and preferences for UGS is crucial. Prior studies applied self-report methods to measure emotional responses to stimuli, which only capture “high-order emotions” based on deeper perceived processing of the stimuli with a variety of forms of bias [16,17,18,19,20]. However, with the rapid advancement of facial expression recognition technology (FER), some studies have applied this model to map the interaction between humans and UGS [21,22,23]. In this study, the objective FER approach was employed to collect emotional perception in supplement with subjective aesthetic preference results. Facial expressions reflect instant and valid emotion data when participants view the urban landscape stimuli. Researchers often use two main categories to describe emotions: (1) basic (e.g., happiness, sadness, anger, and fear) and (2) dimensional approaches [24,25]. The two dimensions used to distinguish emotions are valence and arousal. Valence evaluates pleasantness (positive or negative), while arousal indicates the level of emotional activation [26,27]. The face recognition model detects and reads images of the participants’ faces frame by frame after inputting the facial video recordings, classifies them using deep learning techniques, and then outputs emotional perception big data in two dimensions [28,29], which enables the possibility of capturing real-time emotional perception towards stimulation.

In recent years, researchers have used street-level-image-based methods to conduct research in a more human-centric way [30]. Utilizing GSV images [31,32] has been proposed as a valuable library for providing panoramic and street-level urban streetscape environments from the perspective of pedestrians [33]. Classification is essential for obtaining quantitative data on physical properties in GSV-based visual variable estimation. Traditional information extraction methods, such as Adobe Photoshop software, are falling increasingly short of expectations for big data mining [34] since they are inaccurate, easily affected by image quality, and can only delineate the greenery as a whole class [35]. In this study, a state-of-the-art deep learning framework was employed to extract objective physical properties [36] at multiple detailed levels with high accuracy [37,38]. Deep learning models have the ability to automatically learn hierarchical feature representations and have been widely utilized in image classification and pattern recognition [39,40]. The semantic segmentation model was taught by datasets containing a high number of pictures, allowing for the automatic detection of elements such as grass, buildings, and sky in the scenes, which facilitated it to calculate the changing visual variables of UGS.

In this study, deep learning models were used in tandem to capture accurate and valid emotional perception data and extract detailed variables of the percentage of landscape elements from stimulation in real time. Furthermore, we took video-simulated British Heritage landscapes as a case study, and we obtained changing visual variables and corresponding emotional responses in a controlled setting. The following research topics were explored in this study: (1) the feasibility of this novel quantitative research methodology for instant sentimental assessment of UGS; (2) real-time emotional perceptions towards changing visual variables in a scene; (3) prediction models of public perception with different sets of finer visual variables; and (4) the relationship between FER technology, self-report survey, and body sensor measurements and their distinctions.

## 2. Materials and Methods

### 2.1. Site Selection

For primary stimulation, non-fragmentary landscapes were selected to ensure that each landscape element was distributed in a concentrated and continuous manner to highlight the influence of visual variables. With grand architecture, expansive grass, and lakes, the British Heritage landscape satisfies these requirements. The British landscape stimulation was chosen from the National Heritage List for England (NHLE), which is the United Kingdom’s official list of buildings, monuments, parks and gardens, wrecks, battlefields, and World Heritage Sites.

To maintain emotional levels and avoid emotional declines while watching the primary stimulation with a similar landscape throughout the experiment [25], scenes with a strong contrast with the main stimulus were interspersed as auxiliary stimulation. For auxiliary stimulation, Japanese landscapes with considerable fragmentation and radically different landscaping styles were considered. Similarly, the Japanese landscape was selected from a list of Special Places of Scenic Beauty, Special Historic Sites, and Special Natural Monuments designated by The Minister of Education, Culture, Sports, Science and Technology (MEXT) of Japan under the Law for the Protection of Cultural Properties.

### 2.2. Stimulation Generation

**Figure 1:**
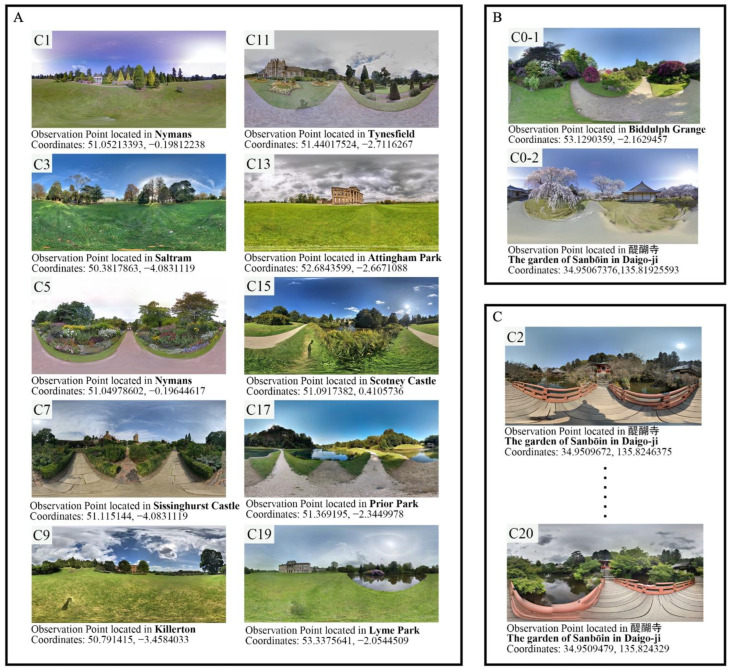
Panoramas taken from the selected observation points were used to create (**A**) primary stimulation, (**B**) practice trials, and (**C**) auxiliary stimulation. The first and final panoramas of (**C**) auxiliary stimulation were captured at a location that was nearly identical but during a different time of year. The “CX” symbol in the upper left corner of each panorama, which is a reference to Table 3, indicates the display order of the associated video clips.

The procedure begins with downloading GSV photos from Google Maps using the GSV Application Program Interface (API) key via Street View Download 360 Pro (version 3.1.3) software (Thomas Orlita, United Kingdom). The collection and analysis of network behaviour data, such as community-driven hashtags, which are ubiquitous and adaptable annotations of public data, has become a new tool to research public preferences in the era of big data [41]. In line with the Instagram hashtag ranking, heritage was scanned by popularity, and the most representative panoramic views of each heritage were selected. Following a series of filters, primary and auxiliary stimulation observation points were selected in the heritage sites listed in Table 1, and the panoramas are presented in Figure 1. Only high-definition panoramas shot under clement weather conditions and from the typical observation angle with the best view were included, while some properties should be discarded due to a lack of images of a specific location, poor weather, or low resolution.

**Figure 2:**
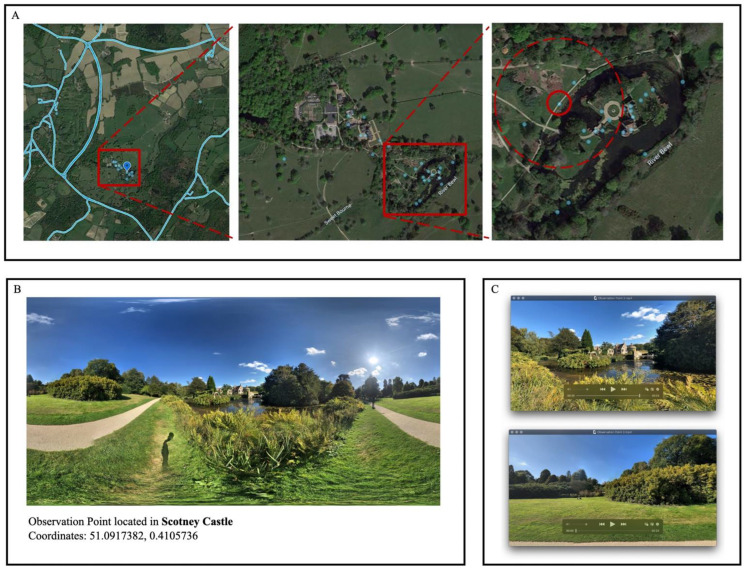
Panoramas from millions of shooting locations are available on the GSV platform. Shooting locations are continuous along the roads, as shown in (**A**), and appear as a blue line, whereas they are mainly dispersed in the off-road area, where they appear as blue dots. (**B**) Use the Street View Download 360 Pro (version 3.1.3) program to download a specific panorama. (**C**) Create a panoramic video clip using the specified parameters after converting the panorama. Take C15 as an illustration.

After downloading the GSV photos, a software project transformed the panoramas into panoramic video clips to imitate a human-centric vision. The following parameters were used: size = 1920 × 1080, FOV (field of view) = 70, pitch = 0, frames per second (FPS) = 30, length = 24 s per clip, spin direction = clockwise (primary stimulation) or anticlockwise (auxiliary stimulation). The stimuli are generated at a reasonably high bitrate in high definition, with adjacent videos spun in reverse to lessen the disorientation that can occur when the video clip is rotated. In this scenario, panoramic video clips can provide participants with a more immersive, comprehensive, and realistic experience of viewing the area from the intended viewpoint than pictures (Figure 2). In the actual experimental process, panorama video clips can be freely generated and mixed in accordance with varied experimental designs.

### 2.3. Segmentation of Primary Landscape Elements

**Figure 3:**
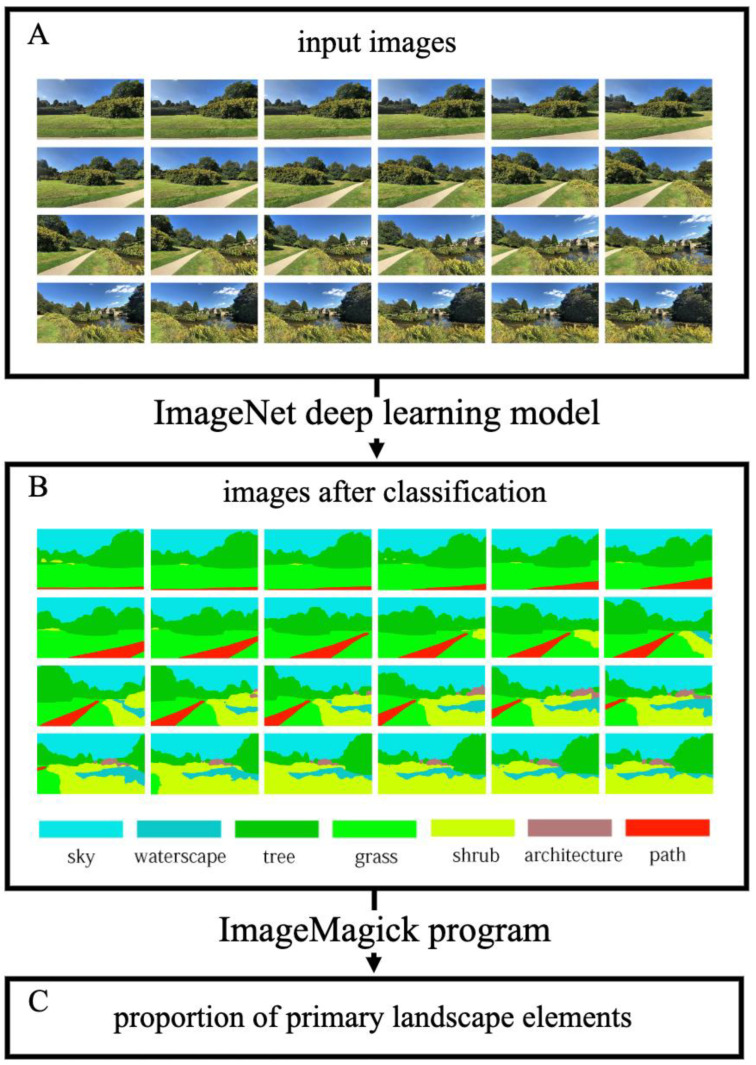
An example of how dominant landscape elements can be extracted along with their proportional data from a scene. Use C15 as an illustration and sample once every second. First, (**A**) frame images are given to the ImageNet deep learning model. Second, (**B**) acquire the classified pictures. Third, count the number of pixels in each class using the ImageMagick program, and (**C**) obtain the proportion information.

For region proposal and feature extraction, we employed the ImageNet detector with the PSPNet-101 backbone for each frame of main stimulation. ImageNet is a deep convolutional network architecture developed for pixel-level semantic segmentation and built on top of the PSA deep learning library [39]. ImageNet outperformed previous algorithms for scene segmentation in more detailed classes and was more computationally efficient. The ADE20K, PSACAL VOC 2012, and Cityscapes datasets were used to pretrain the detectors. We chose the model pretrained on ADE20K for this study because it divides a scene image into 150 detailed classes as opposed to the 21 and 19 classes of the other two models. For example, the Cityscapes model only classifies greenery into one vegetation class, whereas the ADE20K model classifies trees, grasses, and shrubs individually. In our framework, we adjusted the model conforming to the experimental requirements and then obtained seven detailed classes (Figure 3). Researchers had to compile the code and write a batch script in Python to batch process thousands of photos.

**Figure 4:**
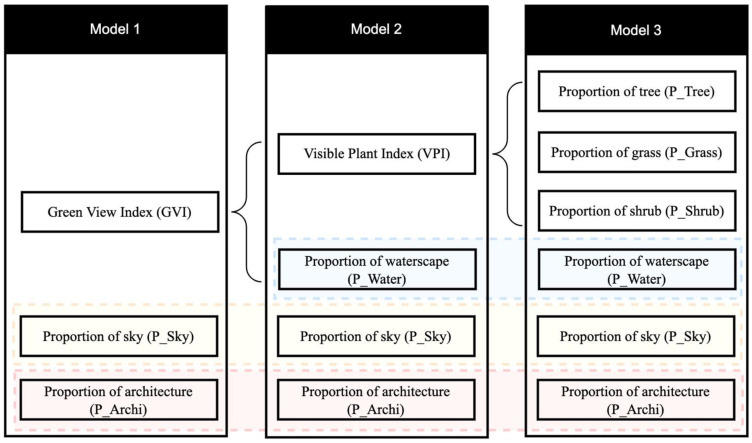
The containment relationships among the eight objectively measured variables. The three backwards multiple linear regression combinations of the eight independent variables.

After classification of stimulation, we used the ImageMagick program to count the number of pixels in each class with a unique colour. The pixel results allow for precise calculation of the visual variables of various primary landscape elements. The framework is also scalable to quantify attributes of space quality, such as space openness and building closure. In this study, eight objectively measured variables of UGS are studied, including green view index (GVI), visible plant index (VPI), proportion of tree (P_Tree), proportion of grass (P_Grass), proportion of shrub (P_Shrub), proportion of waterscape (P_Water), proportion of sky (P_Sky), and proportion of architecture (P_Archi). The containment relationships among the eight variables are shown in Figure 4.

### 2.4. Acquirement for 5 Million Sentiment Data

To capture participants’ immediate emotional perceptions, we adopted the AffectFace dataset and available resources on the ABAW website to retrain the deep learning model for FER. Small samples are common in studies applying physiological techniques (e.g., [24,25,26]), and we recruited 50 healthy participants and gathered valid datasets from 42 of them, resulting in five million big data points for analysis. The mean age of the participants was 23.4 years (SD 1.5, minimum 20 years, maximum 27 years). See Table 2 for sociodemographic information. To ensure a specific degree of emotional awakening, participants should all be at a similar level of unfamiliarity with the landscape in primary stimulation. To avoid different cognitive backgrounds, participants were selected from the population of Chinese college students who lived in China before the age of 15 and had no background knowledge of systematic planning and tourism. Participants were also excluded if they had ever visited the United Kingdom or had any history of mental illnesses or eye diseases.

### 2.5. Laboratory Setting

Potential difficulties linked to the laboratory setting that may affect the results are examined in advance based on previous experience and study [24]. The laboratory was clean and comfortable, with a consistent temperature. Since FER data collection is sensitive to light changes, all lights were kept on throughout the experiment to maintain a stable and homogeneous laboratory environment for emotion tracking. We used a 32-inch 1800R curved-screen monitor to play target videos and a Canon PowerShot G7 X Mark II Digital Camera (Canon, Tokyo, Japan) to capture the expressions to acquire the most realistic portrayal of emotion. The investigator sat directly behind the monitor to avoid eye interference to participants and used MacBook Pro (Apple Inc., Cupertino, CA, United States) to control the video progress through the HDMI cable. To avoid eye contact with participants, the investigator sat directly behind the monitor and utilized a MacBook Pro to manage the video progress via HDMI cable. All experiments were conducted in the same laboratory room with the same settings.

### 2.6. Procedure for Aesthetic Emotion Tracking

When participants arrived at the testing lab, they were asked to take a seat in front of the monitor, approximately 60 cm away, with the centre at eye level. The researcher chatted with the participants to put them at ease and then briefed them on the procedure and the issues that needed to be addressed. Participants were then instructed to settle down and feel their own pulse for 60 s after completing the background questionnaire.

After preparation, participants watched the pre-set stimuli in random order (see one of the random orders in Table 3). Participants conducted practice trials after a ten-second white blank screen to become used to the process. Participants were invited to observe specific landscape panoramic videos and provide a score between zero and ten for their overall aesthetic preference for the scene at the end of each video when the white blank screen appeared. The white blank internals between videos were intended to guarantee that the previous video had no effect on the emotions evoked by the subsequent video. After the rating was completed, researchers controlled and began playing the following video clip.

**Figure 5:**
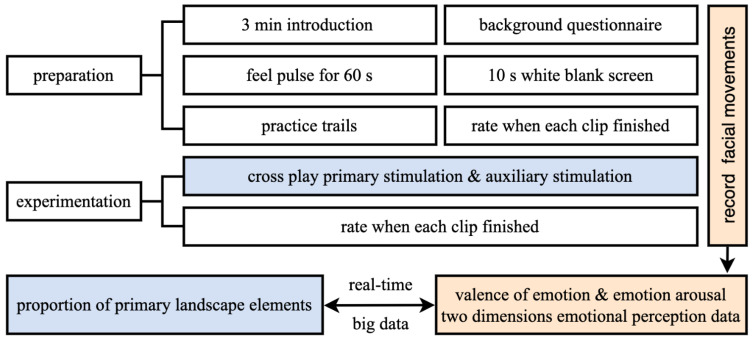
Procedure of preparation and experimentation.

Following the practice trials, participants began the main experiment. Primary and auxiliary stimulation were cross-played. Except for practice trials and auxiliary stimulation, each participant viewed the primary stimulation in a different random order. Participants watched and rated all the panoramic video clips at their own pace (Figure 5).

### 2.7. Analysis

For data cleaning, we sampled one frame per second uniformly and extracted region features for both emotional perception and objectively assessed visual variables data. Unfortunately, because the video clips were produced at a high bit rate, approximately six participants claimed that the video paused occasionally when C3 was played, leading to a nonsensical negative reaction. Consequently, C3 data were eliminated from further investigation.

For the research question of this paper, the valence and arousal dimensions of emotional data, aesthetic preference, and the dominant visual variables of UGS were investigated. Descriptive statistics, summary t-tests, paired t-tests, correlation analysis, and regression analysis were all performed with SPSS. The extensive Matplotlib library was used to process the overall visualization in Python. Pearson’s r correlations were calculated to investigate the correlations between visual variables of dominant landscape elements and public emotional response. Then, backwards multiple linear regression analysis was performed with the valence and arousal emotion dimensions and rating scores as the dependent variable and the proportion of dominant landscape elements in a scene as independent variables. Because it is hypothesized that the explained variance for more detailed sets of visual variables is likely to be higher than for all-inclusive variables, the linear regression was analysed independently for different sets of visual variables. Furthermore, paired t-tests were calculated to study the possible emotional responses elicited by the amount of green in a scene. Finally, the measurements of public perception were studied to evaluate how aesthetic preferences relate to the two major dimensions of emotion.

## 3. Results

### 3.1. A Novel Approach for Gaining Real-Time Emotional Evaluation towards Primary Stimulation

In this study, we innovatively combined two deep learning models to obtain one-to-one corresponding big data in the unit of the frame. First, for pixel-level segmentation analysis, we input panoramic video stimulation into the deep learning model. The visibility of the primary landscape elements viewed from one of the observation points from a visitor’s panoramic perspective is represented by the continuously varying visual variables of the landscape. The variables’ values were precisely calculated for each frame of the generated stimulation, totalling half a million data points. Figure 3 illustrates the segmentation process, and Table 4 lists the results of eight variables for all primary stimulations.

**Figure 6:**
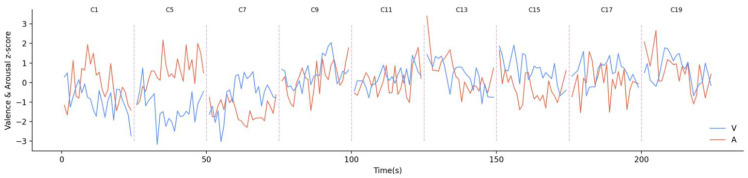
Real-time changes of emotional perception data.

Second, we recorded participants’ facial expressions as they watched the stimulation and adopted another deep learning model to analyse the recorded facial video and obtain the continuously changing emotional data in the unit of frame. The model output more than five million emotional big data points as a result of the approach. The standardized results based on a z score are given in Figure 6 with the participants’ real-time valence and arousal emotional data from primary stimulation. Under the cross-play form, the volatility indicated that participants’ emotional perception levels did not decline.

### 3.2. Relationship between Visual Variables and Emotional Perception Data

#### 3.2.1. Pearson’s R Correlations

To compare visual variables and emotional perception data, Pearson’s r correlations were calculated. Table 4 shows the correlations for all primary stimulations taken together. In general, except for P_Archi, all variables have a significant correlation with the valence (V) value. GVI, VPI, P_Tree, and P_Shrub have negative correlations, while P_Grass, P_Water, and P_Sky have positive correlations. Arousal has a smaller number of correlations than valence. Only P_Grass, P_Shrub, and P_Sky significantly correlated with the arousal (A) dimension. Both V and A found correlations for the variables P_Grass, P_Shrub, and P_Sky.

#### 3.2.2. Predicting Emotional Perception

The significant correlations are further described using backwards multiple linear regression analysis with three alternative combinations of the eight variables as independent variables. Figure 5 depicts the descriptions and containment relationships among eight objectively measurable variables employed by different models. Table 5 displays the significant predictors that emerged from the multiple regressions.

First, we found that visual variables explained emotional perception. The adjusted coefficient of determination (R^2^) of the basic model (Model 1) for V was 0.159 (*p* < 0.01) and for A was 0.098 (*p* < 0.01), indicating that general visual variables were responsible for 15.9% of the variation in the valence dimension and 9.8% of the variation in the arousal dimension of perception data. GVI, P_Sky, and P_Archi were included as independent variables in Model 1. All three variables appear to be significant predictors for emotional perception in this dataset. The strongest predictors are GVI and P_Sky. The weaker predictor is P_Archi.

Second, after separating GVI into VPI and P_Water, we found that our measures of each frame of scene explained additional variance for V. After splitting the indicators of the basic model into more detailed variables, the adjusted R^2^ of the model for V improved to 0.185, indicating that these detailed variables explained 18.5% of the variation in the valence dimension. As a result, the new variables had better explanatory power than the Model 1 variables. Model 2’s adjusted R^2^ for A is the same as that of Model 1 (adjusted R^2^ = 0.098). In Model 2, we used four independent variables: VPI, P_Water, P_Sky, and P_Archi. For both V and A, all four variables appear to be significant predictors. VPI and P_Sky are the best predictors. P_Water and P_Archi are poor predictors.

Third, we continuously divided VPI into P_Tree, P_Grass, and P_Shrub and used six detailed variables, P_Tree, P_Grass, P_Shrub, P_Water, P_Sky, and P_Archi, as independent variables. The variation in V increased again after reseparating the indicators into more precise variables. The adjusted R^2^ of the model for V increased sharply to 0.295, suggesting that the most thorough set of variables explained 29.5% of the variation in the valence dimension. Furthermore, the adjusted R^2^ of the model for A climbed to 0.130. The detailed variables in Model 3 consequently had the strongest explanatory power of the three models’ variables. The other four variables, apart from P_Tree and P_Shrub, appear to be significant predictors of V. P_Grass and P_Water are the best predictors for V, whereas P_Sky and P_Archi are the worst. Unlike V, each of the six variables appears to be a substantial predictor of A. P_Grass, P_Shrub, P_Tree, and P_Sky are the strongest predictors for A, while P_Water and P_Archi are weaker predictors.

### 3.3. Real-Time Emotional Evaluation of Different Amounts of Green in a Scene

**Figure 7:**
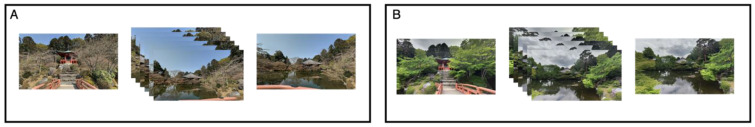
Sample frame images of (**A**) C2 and (**B**) C20. C2 and C20 are panoramic video clips of almost the same observation point in H15. C2 is the winter vision and C20 is the summer vision.

To research how people emotionally react to the amount of green in a scene, we added panoramic video clips of roughly the same observation point in different seasons as auxiliary stimulation (Figure 7). Since the pair of scenes must be observed from the same location, we placed the two video clips at the beginning and the end of the stimulation video set to avoid the familiarity of participants (C2 & C20). In the case that there is a decline in emotion when viewing a similar scene the second time, the winter version was played ahead with the assumption that it may arouse more negative emotions than the summer version.

Including FER emotional perception and aesthetic preference, the descriptive statistics results are shown in Table 6. As Table 6 shows, the findings of V (N = 1008) and A (N = 1008) are higher than those of aesthetic ratings (R, N = 42) after the identical experimental approach. While ratings only disclose the overall landscape preference for the entire clip, FER perception data can reflect the emotional fluctuation of each frame of the participant. These findings support the notion that FER appears to be better at detecting subtle emotional responses than self-report methods, as also demonstrated in Appendix A.

To determine whether there is a general difference in perception for low-green and high-green clips, we conducted a paired t-test between the perception results of C2 and C20. As shown by the paired samples test results, there were significant correlations between the emotional responses to the two versions in V (Correlation = 0.766, *p* < 0.01) and A (Correlation = 0.689, *p* < 0.01). In general, participants reported higher perceived values for high-green clip than for low-green clip. When comparing the high-green clip to the low-green clip, there was a significant increase in R (df = 41, t = 4.27, *p* < 0.01) and V (df = 1007, t = 8.74, *p* < 0.01) but no significant difference in A (df = 1007, t = 1.05, *p* > 0.1) (Table 6).

**Figure 8:**
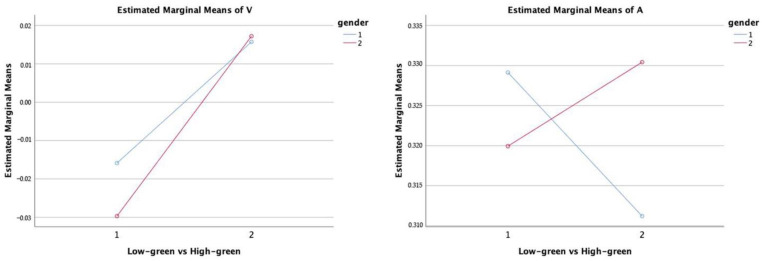
The gender differences in the perception of different amounts of green in a scene. The “Low-green vs. High-green” indicates two scenes: (1) low-green; (2) high-green. The “gender” indicates two genders: (1) men; (2) women.

The gender differences in the pairs of clips were further investigated by performing descriptive statistics, summary t-tests and paired t-tests, with the results reported in Table 7. When the amount of green differed, men (t_R_ = 3.03 *; t_V_ = 4.39 **) and women (t_R_ = 3.39 **; t_V_ = 7.75 **) both reported significant increases in R and V when viewing the higher-green clip. Men (t_A_ = −3.27 **) reported significant declines in A, whereas women (t_A_ = 3.11 **) reported significant rises, resulting in men reporting considerably lower perceived values in the arousal dimension for the low-green condition (t = −2.45 *). In the valence dimension, women increase more dramatically than men, indicating that women are more sensitive to green than men (Figure 8). In V (Dif. = −87% and −10%), there was a huge gender difference (i.e., mean difference in men’ and women’ scores), showing a significant difference in emotional perception between men and women in the low-green condition, while perceptions became similar in the high green condition. However, because it was a comprehensive evaluation, gender differences in green perception could not be distinguished by self-reported aesthetic preference (Dif. = 2% and 1%), confirming that real-time emotional assessment can accurately capture subtle and short-lived emotional swings and allow differences between respondents to be reliably assessed.

### 3.4. Relationship among Measures of Perception

To study the relationship between different measurements, we used the maximum, average, or minimum results of participant’s emotional data toward each video clip to construct Pearson’s correlations among the six calculated emotional perception results (Vmax, Vave, Vmin, Amax, Aave, Amin) and R. The results in Table 8 suggest that only the maximum valence result of each clip has positive significant correlations with aesthetic preference (*p* < 0.01), illustrating that “Vmax” could primarily reveal public perception judgements.

## 4. Discussion

### 4.1. Emotional-Oriented Dynamic Landscape Assessment Framework

Robust evidence is critical for policy-makers and urban planners, as urban development is time-consuming and costly [33,42]. In this paper, we approached the issue from a big data perspective by first proposing a quantitative research framework and demonstrating its feasibility for the continuous emotional assessment of UGS. By applying two deep learning models together, physical features of stimuli and participants’ emotional reactions were extracted accurately and efficiently, ultimately producing five million pieces of big data. The framework can be utilized for UGS dynamic assessment anywhere GSV images are available, and it is adaptable to any experimental design for other computed spatial quality properties.

For stimuli generation, we obtained GSV panoramas of the alternative properties that receive much public attention in the manner of hashtag ranking, which might help prevent cognitive biases caused by the controversial nature of the landscape. Scenic panoramas were first converted into panoramic video clips and then generated to provide experimental stimuli in our procedure. The first step in acquiring quantitative information of each variable was to classify primary landscape elements from panoramic video clips frame by frame. To segment primary landscape elements into different classes, this study used ImageNet trained on the ADE20K dataset. Compared to traditional methods, ImageNet achieved higher scores for scene segmentation in more detailed classes with improved computational efficiency and accuracy, allowing for the linear regression of more detailed sets of visual variables rather than using all-inclusive variables such as GVI. Accordingly, the researchers did not need to gather the hundreds of questionnaires that a self-reported study would ordinarily necessitate.

Moment-to-moment measurements were taken each frame for emotional perception data, allowing variations between respondents and short-lived emotional changes to be reliably assessed. Since the visual stimulation was well set, the researcher was able to determine which frame the observers were observing when they produced a subtle expression change through time nodes to match the emotional data with the objective variables of UGS one by one. Everyone viewed the same video clips of panoramic scenes at the same speed in a laboratory setting. Compared to field observation, a slight bias caused by participants’ varied view angles [43] can be eliminated using this strategy.

### 4.2. Comparison of Real-Time FER Technology, Self-Report Survey, and Body Sensor Methods

Objective measurements of emotions such as facial expression recognition (FER), skin conductance (SC), and facial electromyography (EMG) have been widely used in recent decades to be consistent with self-reported and post hoc interview results and to be able to better distinguish between different dimensions of emotion [18,25,44]. All of the methods listed are capable of accurately recording dynamic and short-lived emotional changes [44], but only FER allows people to feel free during an experimental laboratory setting because it detects subtle and instant expression differences from facial muscle movements recorded by camera, whereas other methods require attaching a sensor, such as an electrode, which may interfere with participants’ natural reactions [25,45]. 

The self-report score only reflects the aesthetic preference for the entire clip [46], while the emotional evaluation is subconscious and non-discrete and occurs in real time [19,24,26]. Self-reported surveys using questionnaires are straightforward to administer, but they have been criticized since the interval between when perceptions are elicited and when participants report them may result in recall inaccuracy and may not be representative of the emotions experienced [18,25]. FER was employed to capture initial emotional reactions while assessing emotional responses in a more relaxed state than other psychophysiological methods [18]. Using FER to capture moment-to-moment emotional responses that are not disclosed by self-report methods can avoid retrospective reflection and cognitive bias [47,48]. FER may clearly be used cooperatively to provide a better and more accurate understanding of emotional experiences by extracting reliable and valid emotion data from participants [18,24,25,45]. Because emotional perception results track minor emotional reactions and distinguish changes promptly and correctly, it is possible to examine emotional evaluation with just a few clips.

FER emotional perception refers to short-lived and unconscious emotional responses to stimuli, while self-reported aesthetic preference relates to the overall view of a scene. The valence dimension refers to pleasant sentiments, and it is worth mentioning that aesthetic preference is significantly related to the scene’s maximum valence result. This suggests that if there are several frames of scene in the clip that give people more pleasure, the overall scores of aesthetic preference may be higher. Thus, the maximum result of valence of each scene can mainly reveal perception judgements to some extent.

One notable outcome is that the real-time FER aids in the detection of minor variations that are difficult to distinguish from aesthetic preferences. The huge valence disparity demonstrated that women were more sensitive to changes in the amount of green than men, implying that women were more likely than men to experience pleasure when watching scenes with higher greenness. However, there was essentially no difference in aesthetic preference between men and women. As a result, FER was able to capture the differences more easily than self-report surveys, emphasizing the importance of applying FER techniques in supplement with self-report surveys to provide a real-time assessment and improved understanding of emotional perception.

### 4.3. Relationship between Visual Variables and Emotional Perception

Regarding the changing visual variables of different landscape elements in a scene, participants reported great changes in aesthetic preference and FER emotions. Knowing this, researchers continued to examine the association between the volatility of UGS visual variables and emotional expressions.

When viewing the high-green clip of a nearly identical scene for the second time, participants reported greater perceived values among the various perception results. The amount of green is mostly influenced by trees, which are the most vital landscape elements in urban contexts, and higher tree coverage has a greater function in stress recovery [49,50]. This result replicates previous studies showing that viewing tree canopies can reduce stress and enhance mood while also providing physical, biological, and aesthetic benefits [11,51]. Participants were more emotionally sensitive to the amount of green in a scene and felt more pleased when the green proportion was higher.

Three different combinations of variables were set as independent variables in a backwards multiple linear regression analysis to predict valence and arousal. Finer visual indicators were classified, and superior regression results were obtained for both valence and arousal by applying deep learning algorithms. The proportion of grass for Model 3, which was the same for arousal, was the best predictive variable of the likelihood of valence. This result replicates previous findings that the amount of grass present in an image is positively related to the restoration likelihood [10]. Scenes with a higher percentage of grass have a greater restorative potential for stress reduction, mental healing, and positive emotional responses. Moreover, the proportion of waterscape was the second most important predictor of valence. Waterscape is widely acknowledged as one of the most essential landscape elements in the creation of therapeutic landscapes, and exposure to blue space promotes healing and wellness [12]. However, little research has been conducted on the relationship between waterscape and human well-being. Participants were sensitive to the presence of waterscape in a scene, feeling calm and peaceful, as shown in Table A1, revealing the restorative effect of blue space. The comfort and attractiveness of the landscape are related to the degree of sky visibility, which can explain many emotional shifts [13]. In agreement with our expectations, the proportion of sky was a significant predictive variable in all three models, which confirmed our expectations.

This study attempted to develop a new research framework for investigating the relationship between diverse landscape elements and aesthetic emotions, which could contribute to the assessment and comprehension of UGS. The current selection of visual variables was founded on the assumption that visual properties and emotional perception are linked. Using the innovative framework, future research can explore the perceivable properties related to landscape character.

### 4.4. Limitations and Future Research

There are some limitations to this study. First, the experimental design did not strictly control the sampling ratio of different sexes. The general results were unaffected by the very small sex differentiation in aesthetic preference and arousal and the larger but parallel differences in valence. Research into the relationship between participants’ emotional perception of UGS and their sociodemographic characteristics like gender, age, occupation, and cultural background could be valuable. Second, GSV makes it possible to present the scene of observation points in various seasonal colours [52], which can be an interesting perspective in further research. Third, precision was sacrificed to extract objective physical features in more detail. The segmentation accuracy of the ImageNet model trained on ADE20K was 81.7%, which is lower than the models trained on the PSACAL VOC 2012 (95.5%) and Cityscapes (96.4%) datasets. It is believed that a higher-precision model will emerge with the rapid development of artificial intelligence, and we can further modify it based on the most state-of-the-art deep learning framework. Fourth, although each panoramic video clip lasted 24 s, participants found it challenging to experience a deeper perceived processing of the stimuli when completing a self-reported survey [17]. New research can also be conducted to enhance the current framework. Furthermore, building a 3D model is a method of better controlling various variables of stimuli, and virtual reality could be considered an effective medium to simulate immersive experiences and elicit emotional perception [53,54]. Finally, while presenting the stimulation gives participants a highly realistic observation experience, it is still unable to restore UGS perception. In realistic environments, complex aspects, such as spatial structure [31], vegetation layout [55], and species diversity [56] can influence aesthetic emotions. In the future, greater in-depth study and synchronous collection of real-time data in the built environment will be necessary.

## 5. Conclusions

Emotional perception is an essential component of UGS assessment; however, most research on these themes is broad and lacks a process-dynamic perspective. This study is an early attempt to propose a continuous emotional assessment framework for UGS, which includes facial expression recognition and primary landscape element segmentation. The emotional responses were gained automatically through FER techniques in combination with the self-reported aesthetic preference ratings, while visual variables of stimuli were classified automatically using ImageNet. From a big data perspective, realizing this revolutionary framework takes advantage of new GSV applications as well as synergistic applications of state-of-the-art deep learning models to extract reliable FER emotional perception data and detailed visual variables of UGSs in real time. Our findings show how the changing visual variables can predict emotional perception in public green space. With finer visual variables, better regression results for both valence and arousal were reached using applying deep learning algorithms. After testing, this quantitative research methodology demonstrates its feasibility and efficiency for objective evaluation from a human-centric perspective, indicating that it might be applied as a support tool for feedback investigations of the built environment and urban design analysis. The framework is applicable to any experimental design for other computed spatial quality properties and can be used for UGS dynamic assessment wherever GSV images are available. This study indicated that the novel framework is well suited for obtaining continuous emotional evaluations of UGS, and the findings could inform policy-making and the design process and allow urban planners to gain a comprehensive understanding of public sentiment towards UGS and create UGS that promote emotional health and well-being.

## Figures and Tables

**Table 1:** Selected heritage landscape with the corresponding hashtag data (assessed from Instagram in 4 October 2020).

Heritage No.	Name	Hashtag
H1	Lyme Park	47,000
H2	Scotney Castle	21,000
H3	Attingham Park	20,000
H4	Nymans	19,000
H5	Tyntesfield	18,000
H6	Saltram	17,000
H7	Killerton	12,000
H8	Sissinghurst Castle	10,000
H9	Prior Park	8182
H10	Biddulph Grange	6978
H11	銀閣寺 Jisho-ji Garden	140,000
H12	天龍寺 Tenryū-ji Garden	115,000
H13	六義園 Rikugi-en	112,000
H14	浜離宮恩賜庭園 Kyu Hamarikyu Gardens	51,000
H15	醍醐寺 The garden of Sanbōin in Daigo-ji	49,000
H16	大徳寺 Daitoku-ji Garden	36,000
H17	鹿苑寺 Rokuon-ji Garden	33,000
H18	桂離宮 Katsura Imperial Villa	14,000
H19	水前寺成趣園 Suizen-ji Jōju-en	8286

Note: 1–10 are heritage sites in the United Kingdom and 11–19 are heritage sites in Japan. The observation points are located in these heritage sites.

**Table 2:** Socio-demographic information of participants (%).

Measures	Categories	%
Gender	Men	26.2 (11)
	Women	73.8 (31)
Age	20–24	83.3 (35)
	25–29	16.7 (7)
Race	Chinese	100.0 (42)
Country lived in before 15 years old	China	100.0 (42)
Occupation	Student	100.0 (42)
Education	Undergraduate	14.3 (6)
	Graduate	85.7 (36)
Landscape/urban planning/architecture-related field	No	100.0 (42)
Have been to UK	No	100.0 (42)
Have been to Japan	Yes	16.7 (7)
	No	83.3 (35)

**Table 3:** One of the random orders to display the video clips converted from selected observation points’ panoramas.

Clip No.	Observation Point Located In	
	Primary Stimulation	Auxiliary Stimulation
C1	H4	-
C2	-	H15
C3	H6	-
C4	-	H17
C5	H4	-
C6	-	H12
C7	H8	-
C8	-	H13
C9	H7	-
C10	-	H16
C11	H5	-
C12	-	H19
C13	H3	-
C14	-	H18
C15	H2	-
C16	-	H11
C17	H9	-
C18	-	H14
C19	H1	-
C20	-	H15

Note: Before C1, the participants viewed 2 clips as practice trials (C0-1 and C0-2) to become familiar with the process. The observation point of C0-1 is located in H10, and the observation point of C0-2 is located in H15. Each participant viewed the primary stimulation in different random orders, while the order of practice trials and auxiliary stimulation were fixed.

**Table 4:** Statistical information of variables for all primary stimulation and two-tailed Pearson’s r correlations between variables and emotional data.

	GVI	VPI	P_Tree	P_Grass	P_Shrub	P_Water	P_Sky	P_Archi
Min (%)	32.44	25.07	2.59	0	0	0	3.67	0
Max (%)	96.85	96.85	43.90	57.16	56.80	40.16	45.30	26.02
Mean (%)	62.80	58.54	19.47	24.29	14.77	4.26	29.37	4.27
SD (%)	13.41	14.96	10.58	17.79	19.89	9.43	8.98	7.12
GVI	1.00							
VPI	0.79 **	1.00						
P_Tree	0.77 **	0.60 **	1.00					
P_Grass	−0.03	0.10	−0.04	1.00				
P_Shrub	0.21	0.35 **	−0.05	−0.80 **	1.00			
P_Water	0.18 **	−0.47 **	0.14 *	−0.21 **	−0.25 **	1.00		
P_Sky	−0.77 **	−0.68 **	−0.59 **	0.30 **	−0.47 **	−0.02	1.00	
P_Archi	−0.72 **	−0.50 **	−0.60 **	0.03	−0.08	−0.23 **	0.24 **	1.00
Valence	−0.22 **	−0.30 **	−0.20 **	0.42 **	−0.49 **	0.18 **	0.34 **	0.13
Arousal	−0.03	0.01	0.07	0.32 **	−0.32 **	−0.02	0.15 *	0.02

Note: * ≤ 0.05, ** ≤ 0.01.

**Table 5:** Results of backwards stepwise regression analysis on the relationship between visual variables and emotional data.

Variables	Model 1		Model 2		Model 3	
	Standardized B, t		Standardized B, t		Standardized B, t	
	Valence	Arousal	Valence	Arousal	Valence	Arousal
**GVI**	0.698, 3.738 **	0.895, 4.628 **				
**VPI**			0.710, 3.434 **	1.022, 4.701 **		
**P_Tree**					N.A.	0.624, 3.159 **
**P_Grass**					0.421, 6.823 **	0.864, 3.103 **
**P_Shrub**					N.A.	0.764, 2.157 **
**P_Water**			0.626, 4.541 **	0.584, 4.021 **	0.300, 4.957 **	0.353, 2.170 *
**P_Sky**	0.762, 5.684 **	0.712, 5.126 **	0.716, 5.379 **	0.728, 5.196 **	0.178, 2.854 **	0.519, 2.884 *
**P_Archi**	0.450, 3.636 **	0.491, 3.831 **	0.457, 3.753 **	0.488, 3.810 **	0.148, 2.423 *	0.388, 2.684 **
(**Constant**)	−4.043 **	18.715 **	−4.043 **	18.716 **	−3.831 **	16.794 **
**Model R^2^**	0.171	0.111	0.200	0.114	0.308	0.154
**Model Adj. R^2^**	0.159	0.098	0.185	0.098	0.295	0.130

Note: * ≤ 0.05, ** ≤ 0.01.

**Table 6:** Descriptive statistics and paired *t*-test results of perception data for different amounts of green.

	Low-Green			High-Green			t
	N	Mean	SD	N	Mean	SD	
Ratings	42	5.81	1.27	42	6.86	1.46	−4.27 **
Valence	1008	−2.61	23.81	1008	1.68	21.15	−8.74 *
Arousal	1008	32.23	11.68	1008	32.54	11.61	−1.05

Note: C2 and C20 are scenes viewed from almost the same point in different seasons. C2 = low-green, C20 = high-green. * ≤ 0.05, ** ≤ 0.01.

**Table 7:** Gender differences for perceptions of different amounts of green.

		Men			Women			Dif. (%)	t
		Mean	SD	t	Mean	SD	t		
Ratings	Low-green	5.91	0.94	−3.03 *	5.77	1.38	−3.39 **	2	0.30
	High-green	6.91	1.10		6.84	1.60		1	0.14
Valence	Low-green	−1.59	17.09	−4.39 **	−2.97	25.77	−7.75 **	−87	0.98
	High-green	1.57	16.96		1.72	22.46		−10	−0.11
Arousal	Low-green	32.91	11.62	3.27 **	31.99	11.70	−3.11 **	3	1.10
	High-green	31.12	10.59		33.04	11.92		−6	−2.45 *

Note: Dif (%) = (perceived value by men—perceived value by women)/perceived value by men × 100. C2 = low-green, C20 = high-green. * ≤ 0.05, ** ≤ 0.01.

**Table 8:** Two-tailed Pearson’s r correlations between ratings and six calculated emotional perception results.

		Valence			Arousal		
		Vmax	Vave	Vmin	Amax	Aave	Amin
Ratings	Pearson’s r	0.137 **	0.094	0.068	0.073	0.100	0.078
	*p*-value	0.008	0.069	0.185	0.157	0.051	0.129

Note: ** ≤ 0.01.

## Data Availability

The data are not publicly available due to the ongoing research, and the authors will continue to work the data in the future.

## Appendix A

The proportion of blue space in contrast to the amount of green remains constant throughout the entire year. Since visual variables in the existing natural and built environments are difficult to regulate, we chose one panoramic video clip and found that the GVI results of the forward half (M = 0.715, SD = 0.011) and the reverse half (M = 0.677, SD = 0.033) were almost the same. Given that waterscape almost completely replaced grass in the reverse part, the proportion of waterscape shows a significant difference between the forward and reverse parts (M = 0.000, SD = 0.000, and M = 0.060, SD = 0.018). Figure 2 and Figure 3 depict the frames of the forward and reverse parts of C15.

The emotional perception results of the study of C15 are divided into the front half and the reverse half because V and A are measured in seconds; however, rating score only reveals the participant’s subjective aesthetic preference for the entire clip. Even though C15 only lasts for 24 s, the deep convolutional network model allows for the collection and calculation of 30,240 emotional datasets and 720 groups of visual variables for all 42 participants. These results provide further evidence that the V and A results are superior to the self-reported approach in identifying mild emotional responses.

**Table A1:** Descriptive statistics and paired t-test results of perception data for different element compositions of similar GVI.

	Forward Half (Grass)			Reverse Half (Waterscape)			t
	N	Mean	SD	N	Mean	SD	
Valence	504	3.96	22.31	504	1.99	22.72	3.28 *
Arousal	504	32.58	13.04	504	32.03	13.40	1.79

Note: The GVI results of C15’s forward half and reverse half are similar but in different element compositions. The area of grass in forward half changed to waterscape in reverse half. Forward half = grass, reverse half = waterscape. Each participant provided a single rating for the whole clip (N = 42, Mean = 6.00, SD = 0.22). * ≤ 0.05.
